# Co-occurrence of Overweight, Stunting, and Anemia among Adolescents (10–19 Years) in Tanzania Mainland: A School-Based Cross-Sectional Study

**DOI:** 10.1016/j.cdnut.2023.102016

**Published:** 2023-10-14

**Authors:** Geofrey Mchau, Erick Killel, Kaunara Azizi, Stanslaus Henry, Samafilan Ainan, Theresia Jumbe, Nyamizi Bundara, Wiggins Kystikila, Felista Mwingira, Pendael Machafuko, Bwire Wilson, Heavenlight A. Paulo, Sauli Epimack, Hoyce Mshinda, Frank Chacky, Ramadhani Noor, Ray Masumo, Germana Leyna

**Affiliations:** 1 Department of Community Health and Nutrition, Tanzania Food Nutrition Centre (TFNC), Dar es Salaam, Tanzania; 2 Department of Epidemiology and Biostatistics, Muhimbili University of Health Allied Sciences (MUHAS), Dar es Salaam, Tanzania; 3 Department of Food Sciences and Nutrition, Tanzania Food Nutrition Centre (TFNC), Dar es Salaam, Tanzania; 4 Department of Pediatric and Child Health, Muhimbili University of Health Allied Sciences (MUHAS), Dar es Salaam, Tanzania; 5 Hellen Keller International (HKI), Tanzania; 6 Department of Human Nutrition and Consumer Studies, Sokoine University of Agriculture (SUA), Tanzania; 7 Ministry of Livestock and Fisheries; 8 Department of Statistics, University of Dar es Salaam (UDSM), Dar es Salaam, Tanzania; 9 Dar es Salaam University College of Education; 10 National Institute for Medical Research, Muheza Branch; 11 National Malaria Control Program, Ministry of Health (MoH), Tanzania; 12 United Nations Children’s Fund (UNICEF), Ethiopia

**Keywords:** overweight, stunting, anemia, double burden, triple burden, Tanzania

## Abstract

**Background:**

Evidence on double and triple burdens of malnutrition among adolescents is an essential key to informing policy design, implementation, and tracking progress of adolescent nutritional programs. Tanzania has a scarcity of studies on the double and triple burden of malnutrition among adolescents.

**Objective:**

The aim of this study was to assess the co-occurrence of malnutrition (overweight, stunting, and anemia) among adolescents (10–19 y) in mainland Tanzania.

**Methods:**

A school-based cross-sectional study was conducted among 44,120 primary school adolescents aged 10 to 19 y in mainland Tanzania. Anthropometric assessments (weight, height, and body mass index), dietary assessments, and hemoglobin levels were used to calculate the single, double, and triple burden of malnutrition. Data were analyzed using Stata software 15. The chi-square test was used to test the association between the nutrition condition and social demographic variables, physical activity, and dietary quality. Log-binomial models were used to determine factors associated with stunting, overweight, and anemia. Multivariable log-binomial models were used to control confounders. All analyses were 2-tailed, and the significance level was set at 5%.

**Results:**

The prevalence of anemia was 34.1%, while stunting and overweight had a prevalence of 32% and 4.2%, respectively. Approximately 41.7%, 13.5%, and 0.3% had single, double, and triple burden malnutrition-related conditions, respectively. Females were found to have a higher risk of being overweight compared with males (relative risk [RR]: 1.33; 95% confidence interval [CI]: 1.21, 1.45), while engaging in moderate to low levels of physical activity was associated with a decreased risk of being overweight. Additionally, residing in urban areas was linked to a decreased risk of stunting (RR: 0.78; 95% CI: 0.75, 0.80) and a 27% lower risk of anemia when compared with participants from rural areas.

**Conclusion:**

The findings from this study suggest that the complex nature of malnutrition among school adolescents warrants consideration when designing policies and interventions to reduce the burden of malnutrition.

## Introduction

A common misconception about malnutrition in low- and middle-income countries (LMICs) is mostly refers to undernutrition (i.e., stunting and wasting). The coexistence of multiple forms of malnutrition (overweight and undernutrition [i.e., stunting/wasting]) is slowly becoming a prominent health topic in the global health community [1]. In LMICs, however, information on nutritional indicators among adolescents is scarce compared with information on maternal and child nutrition. For instance, in Tanzania, as one of the developing countries, most surveys cover females of reproductive age and children under 5 y old [2,3]. It is well known that being underweight or overweight during adolescence can have serious implications for proper development, survival, the prevalence of chronic illnesses, the economic productivity of individuals and communities, and increased burden on healthcare systems [4]. The effect of adolescents having multiple nutritional conditions concurrently cannot be underestimated. To be specific, overweight in adulthood is widely recognized as a major health obstacle and a significant factor influencing numerous chronic diseases, psychosocial difficulties, and increased risk of noncommunicable diseases [5]. Likewise, anemia among adolescents is a global health problem. However, evidence regarding its burden and risk factors, particularly for younger adolescents in sub-Saharan Africa, remains scarce [5]. This calls for more surveys specifically for adolescents to bridge the gap that would inform targeted interventions for anemia control. Lastly, stunting remains prevalent in LMICs [6]. The consequences of stunting in individuals include diminished cognitive and physical growth and development, reduced productive capacity, and increased risk of degenerative diseases, effects that can appear at present and later in life [7].

Tanzania, like any other developing country, is experiencing a gap in the availability of robust national representative data on adolescent nutrition. The midterm review of the national multisectoral nutrition action plan of 2016/2017 [8] reveals the need for the country to start bridging the gap of adolescent information. According to 2021 population projections, school-aged children, including adolescents, constitute 31% of the total population in mainland Tanzania [9]; this provides a valuable second window opportunity to address nutrition challenges among adolescents that will have significant physical and cognitive implications throughout their life course.

This study aimed at assessing the co-occurrence of malnutrition (overweight, stunting, and anemia) among adolescents (10–19 y). Moreover, the present study bridges the gap of adolescent nutrition information existing within the country, and the results will provide an opportunity to inform policy design, implementation, and tracking progress of adolescent nutritional programs in Tanzania. To attain this objective, a synergistic effort was established among the National Malaria Control Program, the Nutrition section at the Ministry of Health, and the Tanzania Food and Nutrition Centre to include nutrition indicators for the first time in 2019 School Malaria and Parasitological Survey (SMPS), which is conducted every 2 y in mainland Tanzania.

## Methods

### Study area and design

The study was an analytical cross-sectional survey designed to collect information from public primary school adolescents in Tanzania’s mainland covering all 26 regions and 184 councils.

### Study population and eligibility criteria

The target population for this study was all adolescents aged 10 to 19 y in public primary schools. Within some pastoral regions of Tanzania, it is a frequent phenomenon for adolescents to begin their primary education at a later age. This tendency can be attributed to the responsibilities of assisting their families with livestock-keeping during their scholastic journey. Also, grade repetition can contribute to children being over age for their designated grade level. All enrolled adolescents aged between 10 and 19 y present during the day of the survey were eligible to participate in the study as part of SMPS and agreed to participate in this study. Adolescents who were not willing to participate in the study, those with health conditions such as sickle cell, or those under medication during the survey were excluded.

### Sampling procedure and sample size determination

#### Sampling procedure

The study employed a 3-stage multistage cluster sampling. The first stage involved selection of wards clustered within each stratum (council). The second stage involved the selection of a school from a list of schools within a selected ward. The third stage involved the selection of pupils from each selected school using systematic sampling, where a list of all eligible participants was provided to investigators and used to select every *K*^*th*^ participant to be included in the study. Due to disproportional council sizes, probability proportional to size influenced the number of students selected from each council to the school level.

#### Sample size determination

The sample of school pupils was estimated at council level based on council stunting prevalence estimated from the 2017 SMPS [10], 0.05 margin of error, 5% significance level, council population of 10 to 19 y old children, and design effect of 2.5 to account for existing stunting heterogeneity at the council level. The estimated council sample was aggregated to obtain national representative sample of around 44,120 pupils from 661 schools in 26 regions of mainland Tanzania.

### Data collection procedure

#### Social demographic information

To gather social demographic information, pretested semistructured questionnaires were utilized. The questionnaires were designed to capture key social demographic information, including age, sex, education level, household income, and place of residence. The questionnaire tool underwent rigorous pretesting to ensure its validity and reliability in the context of the study. The social demographic information collected was critical to identifying patterns and trends related to overweight, stunting, and anemia in adolescents in mainland Tanzania.

#### Anthropometric assessments

Duplicate anthropometric measurements were collected using SECA weighing scales and ShorrBoard height-length measuring boards to minimize measurement errors. Measurements obtained (height and weight) were used to calculate *z*-scores, which were used to classify individual adolescents as, stunted [height for age z-score less than −2 standard deviations (SDs)], based on the WHO standard [11]. An individual was classified as overweight if the BMI for age *z*-score was above 1 SD and as overweight if the BMI for age *z*-score was greater than 2 SDs, whereas an individual was regarded as thin if the BMI for age *z*-score was below −2 SDs and severely thin if BMI for age *z*-score was less than −3 SDs.

#### Dietary assessments

Additional information was collected to identify factors associated with nutritional status and anemia among adolescents. To assess diet quality, the consumption of a variety of food groups at least once per week was assessed using the Diet Quality Score [12] scale. Items included in the subscale were chosen from the study based on Lazarou et al. [13] and Feskanich et al. [14]. The dietary quality score was categorized by tertile (3 groups with equal proportions) [15]. A score of 1 was given if an individual consumed food from specific food groups 5 to 7 times per week.

#### Physical activity level assessment

To assess the physical activity level (PAL) of study participants, we employed the short version of the International Physical Activity Questionnaire [16] in line with standard procedures. The reliability score of this questionnaire was high (α < 0.80) and valid. The questionnaire was used to determine the frequency, duration, and intensity of physical activity among participants, with a focus on sedentary behavior and moderate to vigorous physical activity (MVPA) as recommended by the WHO.

Using the data collected, participants were categorized into 2 groups, namely, those with sedentary behavior and those engaged in MVPA. This categorization was based on the WHO guidelines for assessing PALs. The results of the assessment were important in understanding the participants’ PALs and their relationship with overweight, stunting, and anemia in adolescents in mainland Tanzania.

#### Blood sampling and analysis

A blood sample was obtained via finger-pricking following disinfection of the fingertip with a swab containing 70% isopropanol (Heinz Herenz). The first drop of blood was removed with a sterile cotton swab, the second drop was collected for a rapid diagnostic test for malaria investigation, and the remaining blood was collected into HemoCue micro cuvettes. Hemoglobin concentration was determined photometrically using the HemoCue Hb 201 analyzer (HemoCue). Readings were documented immediately and adjusted for altitude in specific localities during analysis. The severity of anemia was defined based on WHO cutoff points [17], as shown in Table 1.

**TABLE 1:** Anemia cutoff points for adolescents in Tanzania’s mainland

Anemia status^1^	Hb level (g/dL)		
	School adolescents aged 10–14 y^2^		School adolescents aged 15–19 y
	Age 10–11 y	Age 12–14 y	
Any anemia	<11.0	<12.0	Girls: <12.0
			Boys: <13.0
Mild	11.0–11.4	11.0–11.9	Girls: <11.0–11.9
			Boys: <11.0–12.9
Moderate	8.0–10.9	8.0–10.9	8.0–10.9
Severe	<8.0	<8.0	<8.0

Hb, hemoglobin

Table 1 above was adapted from WHO [17].

1 Individual Hb values were adjusted for altitude greater than 1,000 m above sea level.

2 According to Hb concentration for diagnosis of anemia and assessment of severity, WHO, 2011: Cut-off for adolescents aged 10 to 11 years are different from those aged 12 to 14 years.

### Statistical analysis

Data were cleaned and analyzed using Stata version 15 (2015 Stata Statistical Software: Release 14, StataCorp LP). Categorical variables were analyzed using frequency and proportion and presented in figures and narrations. The chi-square test was used to test the association between the nutrition condition and social demographic variables, physical activity, and dietary quality. Log-binomial models were used to determine factors associated with stunting, overweight, and anemia. A multivariable log-binomial model was used to control confounders. All analysis was 2-tailed, and the significance level was set at 5%.

In this study, we categorized as single, double, and triple burden of malnutrition. Double burden was defined as the co-occurrence of 2 forms of malnutrition (undernutrition [stunting], overnutrition [overweight], or anemia), and triple burden was defined as the co-occurrence of all 3.

## Ethical approval

Ethical clearance was sought from the National Health Research Ethics Committee of the National Institute for Medical Research before the implementation of the survey. Written consent was obtained from parents and affirmative assent from the participants themselves. Invitations to take part in the study were sent out with written information detailing the research and information regarding their rights, safety, and confidentiality as research participants.

## Results

A total of 44,120 adolescents participated in the National School Malaria and Nutrition Survey in Tanzania in 2019. Both males and females were equally represented, with 51% being male and 49% being female. The majority of participants (90.4%) were aged between 10 and 14 y, with half of them being male. About three-quarters (74.4%) of the participants lived in rural areas, and 37.7% of them reported consuming poor-quality diets.

**FIGURE 1:**
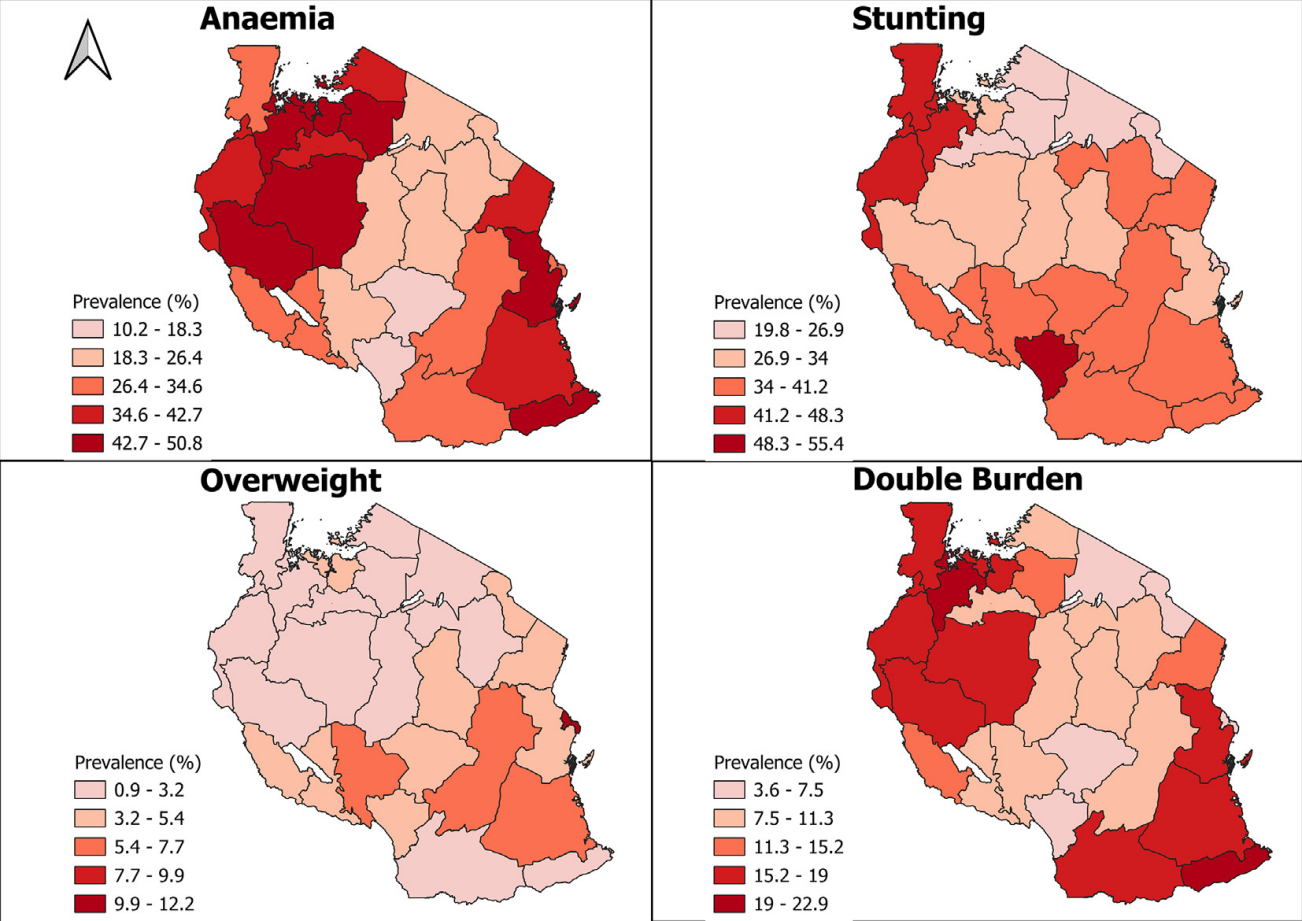
The map of Tanzania mainland showing the burden of malnutrition among adolescents (10–19 y).

The prevalence of anemia was 34.1%, while that of stunting and overweight was 32% and 4.2%, respectively (Table 2). The prevalence of anemia is noticeably high in the western, eastern, and certain parts of the southern zones of Tanzania, specifically in the Mtwara and Lindi regions. Stunting appears to be a significant problem in the southern highlands and northwestern zones of Tanzania. Moreover, overweight is prevalent in certain regions of Tanzania, including Lindi, Morogoro, Dar es Salaam, and Songwe (Figure 1).

**TABLE 2:** Baseline characteristics of randomly selected (*N* = 44,120) adolescents who participated in the National School Malaria and Nutrition Survey in Tanzania, 2019

Background characteristic	Frequency	Percent
Sex		
Male	22,486	51.0
Female	21,634	49.0
Age, y		
10–14	39,863	90.4
15–19	4257	9.6
Residence		
Rural	32,808	74.4
Urban	11,312	25.6
Height for age (HAZ < −2 SD)		
Normal	29,963	68.0
Stunted	14,076	32.0
BMI for age		
Thin	6049	13.8
Overweight	1856	4.2
Normal	36,011	82.0
Anemia status by hemoglobin level^1^		
Anemic	5207	34.1
Normal	10,067	65.9
Dietary Quality Score among participants		
Poor	16,653	37.7
Medium	14,475	32.8
High	12,992	29.5

1 Anemia based on hemoglobin level was assessed in a subsample (one-third of total sample).

About 41.7%, 13.5%, and 0.3% had single, double, and triple burden malnutrition-related conditions, respectively. There was significantly higher burden (single 43% and double 15%) of malnutrition among rural residents (*P* < 0.01). More than a quarter (16.1%) of those with double burden are male. Of those with a single burden, 46.1% had malaria compared with 24.3% in those with a double burden (*P* < 0.01) (Table 3).

**TABLE 3:** Triple/double burden of overweight, anemia, and stunting among adolescents (*N* = 15,274)

Variables	No burden *n* (%)	Single burden *n* (%)	Double burden *n* (%)	Triple burden *n* (%)	*P*
Sex					
Male	3265 (41.7)	3280 (41.9)	1259 (16.1)	27 (0.3)	
Female	3519 (47.3)	3096 (41.6)	803 (10.8)	25 (0.3)	<0.001
Age, y					
10–14	6371 (46.5)	5670 (41.7)	1620 (11.8)	43 (0.3)	
15–19	413 (26.3)	706 (45.0)	442 (28.2)	9 (0.6)	<0.001
Residence					
Rural	4706 (41.5)	4882 (43.0)	1715 (15.1)	41 (0.4)	
Urban	2078 (52.9)	1494 (38.0)	347 (8.8)	11 (0.3)	<0.001
Physical activity					
Sedentary	1994 (43.8)	1934 (42.4)	609 (13.4)	20 (0.4)	
MVPA	4790 (44.7)	4442 (41.5)	1453 (13.6)	32 (0.3)	0.344
Dietary quality					
Low	2506 (43.8)	2408 (42.1)	791 (13.8)	22 (0.4)	
Medium	2219 (44.3)	2084 (41.6)	694 (13.8)	16 (0.3)	
High	2059 (45.4)	1884 (41.6)	577 (12.7)	14 (0.3)	0.520
mRDT results					
Negative	6069 (47.3)	5250 (40.9)	1468 (11.4)	42 (0.3)	
Positive	715 (29.2)	1126 (46.1)	594 (24.3)	10 (0.4)	<0.001
Total	6784 (44.5)	6376 (41.7)	2062 (13.5)	50 (0.3)	

A single burden was defined as the presence of any 1 of 3 predefined conditions. A double burden was characterized by the occurrence of 2 out of the 3 conditions, and a triple burden was defined by the simultaneous occurrence of all 3 conditions. Dietary quality was categorized by tertile (3 groups with equal proportions). Statistical significance set at *P* < 0.05.

Abbreviations: mRDT, malaria Rapid Diagnostic Test; MVPA, moderate to vigorous physical activity.

### Factors associated with overweight, stunting, and anemia among primary school adolescents in mainland Tanzania: adjusted analysis results

In adjusted analysis, high dietary quality was associated with lower risk of overweight (*P* = 0.046). Females had a higher risk of being overweight (relative risk [RR]: 1.33, 95% CI: 1.21, 1.45) compared with males. Participants from urban areas had 2.2 times higher risk of being overweight (RR: 2.20; 95% CI: 2.01, 2.44) compared with their rural counterparts. Moderate to low levels of physical activity were associated with a decreasing risk of being overweight (RR: 0.91; 95% CI: 0.83, 0.99) compared with those with a sedentary lifestyle. Additionally, participants aged 15 to 19 y had a lower risk of being overweight (RR: 0.57; 95% CI: 0.46, 0.72) compared with participants aged below 15 y. Females had less risk of stunting (RR: 0.76; 95% CI: 0.74, 0.79) compared with males. Living in an urban area was associated with decreased risk of stunting (RR: 0.78; 95% CI: 0.75, 0.80). Similarly, participants from urban areas had 27% lower risk of anemia compared with participants from rural areas (Table 4).

**TABLE 4:** Adjusted regression analysis showing factors associated with overweight, stunting, and anemia among primary school adolescents in Tanzania mainland (*N* = 44,120)

Variable	Overweight		Stunting		Anemia	
	aRR (95% CI)	*P*	aRR (95% CI)	*P*	aRR (95% CI)	*P*
Sex						
Male	1		1		1	
Female	1.33 (1.21, 1.45)	<0.001	0.76 (0.74, 0.79)	<0.001	0.95 (0.90, 1.00)	<0.057
Age, y						
10–14	1		1		1	
15–19	0.57 (0.46, 0.72)	<0.001	1.42 (1.37, 1.48)	<0.001	1.65 (1.53, 1.77)	<0.001
Residence						
Rural	1		1		1	
Urban	2.20 (2.01, 2.41)	<0.001	0.78 (0.75, 0.80)	<0.001	0.73 (0.68, 0.78)	<0.001
Dietary quality						
Low	1		1		1	
Medium	0.96 (0.87,1.07)	0.448	1.02 (0.99,1.05)	0.298	0.98(0.92,1.05)	0.566
High	0.89 (0.80,0.99)	0.046	1.00 (0.97,1.03)	0.966	0.95(0.89,1.02)	0.143
Physical activity						
Sedentary	1		—		—	
MVPA	0.91 (0.83, 0.99)	0.045	—		—	
mRDT						
Negative	1		1		1	
Positive	0.57 (0.48, 0.68)	<0.001	1.24 (1.20, 1.29)	<0.001	1.60 (1.50, 1.71)	<0.001

Abbreviations: aRR, adjusted relative risk; CI, confidence interval; mRDT, malaria Rapid Diagnostic Test; MVPA, moderate to vigorous physical activity

## Discussion

This is the first nationwide survey addressing the co-occurrence of malnutrition among adolescents aged 10 to 19 y that benefited from leveraging the approach of integrating nutrition indicators in the SMPS, which is conducted every 2 y. The practicality of integrating multiple programs in most developing countries to maximize limited resources was best illustrated through the combination of malaria and nutrition surveys in mainland Tanzania, which allowed for a single survey to be conducted from the different perspectives and objectives of each program. The objective of this study was to fill the current gap in adolescent nutrition data in Tanzania by reporting on the co-occurrence of overweight, stunting, and anemia among adolescents in school. Several surveys conducted in Tanzania, such as the Tanzania Demographic and Health Survey (TDHS) and the Tanzania National Nutrition Survey, have limited information pertaining to adolescent nutrition as they only cover females of reproductive age and children aged under 5 y.

The study reported a high prevalence of stunting (32%), which is high as compared with the results of another study performed in Kilimanjaro by Shayo and Lawala [18], which reported a lower prevalence of stunting among adolescents at 6%. The prevalence of stunting in the southern highlands and northwestern zones of Tanzania is significantly high, as demonstrated by the TDHS, and has been a persistent problem over the years.

The current study presents a noticeably high prevalence of anemia in the western, eastern, and certain parts of the southern zones of Tanzania, specifically in the Mtwara and Lindi regions, indicating a potential issue with the availability and access to essential micronutrients, such as iron. The prevalence of anemia among adolescents reported in this study (34%) is higher compared with the findings of other studies conducted in Kilimanjaro among adolescents, which indicate a prevalence of anemia of 23.1%. Moreover, the study reveals a significant difference in mean hemoglobin levels between female and male adolescents. The high prevalence of anemia and stunting among adolescents might be contributed to the consumption of low/poor dietary quality, especially during school break time, among other factors [19].

Our study revealed that adolescents with high poor dietary quality have higher rates of overweight and anemia compared with those with better diets. Also, moderate to low physical activity was associated with a decreased risk of being overweight compared with sedentary lifestyles. Though the level of physical activities was not examined, the hectic school academic timetable likely restricted the physical activity of adolescents, meaning they had less time for physical activity outside of school. The study conducted in Babati rural district by Frumence et al. [20] revealed an overall prevalence of overweight among adolescents of 9.2%, with more girls being overweight than boys, which is considerably more than that reported in this study. Similar findings were reported by studies done in Nigeria and Ethiopia by Adebayo et al. [21] and Dagne et al. [22], respectively. To address this issue, further research needs to be done to identify the underlying causes as well as potential interventions to improve the well-being of adolescents in these regions. Additionally, measures need to be taken to reduce the consumption of carbohydrates and promote the intake of fruits and vegetables among adolescents to reduce their risk of being overweight [23].

The present study shows the existence of double and triple burden of malnutrition among adolescents in mainland Tanzania. A large number of adolescents had a single burden of malnutrition followed by double burden and triple burden malnutrition. In addition, the prevalence of double burden was higher among adolescents aged 15 to 19 y compared to those aged 10 to 14 y. Furthermore, the same age group had a higher risk of anemia compared with early adolescents aged 10 to 14 y. A similar finding was found in the study conducted in Zanzibar, which showed that anemia was more common among girls during later adolescence [24]. Our analysis highlights the co-occurrence of the double burden of malnutrition in the western, southern, and eastern zones of Tanzania. This is a significant concern as it indicates a high prevalence of both undernutrition and overweight, which could lead to a range of health complications. It is crucial to develop targeted interventions that address both ends of the malnutrition spectrum to effectively tackle malnutrition.

This study presents several factors that are associated with stunting, overweight, and anemia, such as high dietary quality being associated with a lower risk of being overweight, residence in urban areas associated with high risk of being overweight compared with rural, as well as female sex. The findings from the present study are similar to a study by Mulu Birru et al. [25] in which a higher prevalence of overweight was found among girls than boys and in urban than in rural areas. Similarly, Adamo et al. [26], who compared children from rural and urban Kenya to their counterparts in Canada, reported that while overweight was nonexistent in the rural Kenyan population, urban Kenyan children were anthropometrically similar to their contemporaries in Canada. This suggests a possible nutritional transition in urban areas that may be partly explained by the adoption of more Western lifestyles by urban dwellers in sub-Saharan Africa. These findings again point to an obvious fact that overweight rates in the region are currently comparable and in some instances exceed rates in highly developed countries, where the reported prevalence ranges from 10% in Denmark to 31% in the United States [27]. Furthermore, such prevalence has been increasing with time at an alarming rate.

The major strength of this study is the use of nationally representative, larger samples covering all regions and districts in both rural and urban areas across the country. However, this study has some important limitations. Firstly, all data were cross-sectional in nature, which limits our ability to determine the causal directions of the associations we observed in this study. Another limitation is that this study examined the sample population of adolescents enrolled in public primary schools, excluding those enrolled in private schools and those not attending school.

## Conclusion

This survey demonstrated the key aspect of leveraging resource usage between programs in LMICs where resources are limited. The study highlights a prevalent triple burden of overweight, stunting, and anemia among adolescents in Tanzania. Moreover, physical activities and the provision of a quality diet among adolescents may have some influence on nutritional outcomes in school settings in this critical age and alleviate the implications of poor health across the lifespan. The observed triple and double burden within the country is unequally distributed; hence, it is critical to develop prevention and control measures for malnutrition challenges, bearing in mind this unique distribution, to achieve the desired outcomes.

## Author contributions

The authors’ responsibilities were as follows—GM, EK, KA, FC, RM: developed project concept; HAP, SH: analyzed data; GM, EK, KA: wrote original draft; GM, FC, HM, TJ, WK, RN, RM, GL: review and editing the final manuscript; and all authors: read and approved the final manuscript.

## Funding

Fieldwork activities including orientation of field teams was supported by the Global Fund to fight AIDS, TB, and Malaria (GFATM) through the Tanzania National Malaria Control Program (NMCP). The US President’s Malaria Initiative (PMI) via the US Agency for International Development Okoa Maisha Dhibiti Malaria (cooperative agreement no. 72062118CA-00002) implemented by RTI International under the terms of an interagency agreement with the US Centers for Disease Control and Prevention provided financial support on data management. The United Nations Children’s Fund (UNICEF) provided fund for collection of nutrition indicator and anemia assessment.

## Data availability

The datasets generated and/or analyzed during the current study are available from the corresponding author on reasonable request.

## Conflict of interest

The authors report no conflicts of interest.
